# Pulmonary Fissure Integrity and Collateral Ventilation in COPD Patients

**DOI:** 10.1371/journal.pone.0096631

**Published:** 2014-05-06

**Authors:** Jiantao Pu, Zhimin Wang, Suicheng Gu, Carl Fuhrman, Joseph K. Leader, Xin Meng, John Tedrow, Frank C. Sciurba

**Affiliations:** 1 Imaging Research Center, Department of Radiology, University of Pittsburgh, Pittsburgh, Pennsylvania, United States of America; 2 Department of Bioengineering, University of Pittsburgh, Pittsburgh, Pennsylvania, United States of America; 3 Department of Radiology, The First Affiliated Hospital, Xi'an Jiaotong University School of Medicine, Xi'an, Shaanxi Providence, P.R. China; 4 Division of Pulmonary Allergy and Critical Care Medicine, Department of Medicine, University of Pittsburgh, Pittsburgh, Pennsylvania, United States of America; University Hospital Freiburg, Germany

## Abstract

**Purpose:**

To investigate whether the integrity (completeness) of pulmonary fissures affects pulmonary function in patients with chronic obstructive pulmonary disease (COPD).

**Materials and Methods:**

A dataset consisting of 573 CT exams acquired on different subjects was collected from a COPD study. According to the global initiative for chronic obstructive lung disease (GOLD) criteria, these subjects (examinations) were classified into five different subgroups, namely non-COPD (222 subjects), GOLD-I (83 subjects), GOLD-II (141 subjects), GOLD-III (63 subjects), and GOLD-IV (64 subjects), in terms of disease severity. An available computer tool was used to aid in an objective and efficient quantification of fissure integrity. The correlations between fissure integrity, and pulmonary functions (e.g., FEV1, and FEV1/FVC) and COPD severity were assessed using Pearson and Spearman's correlation coefficients, respectively.

**Results:**

For the five sub-groups ranging from non-COPD to GOLD-IV, the average integrities of the right oblique fissure (ROF) were 81.8%, 82.4%, 81.8%, 82.8%, and 80.2%, respectively; the average integrities of the right horizontal fissure (RHF) were 62.6%, 61.8%, 62.1%, 62.2%, and 62.3%, respectively; the average integrities of the left oblique fissure (LOF) were 82.0%, 83.2%, 81.7%, 82.0%, and 78.4%, respectively; and the average integrities of all fissures in the entire lung were 78.0%, 78.6%, 78.1%, 78.5%, and 76.4%, respectively. Their Pearson correlation coefficients with FEV1 and FE1/FVC range from 0.027 to 0.248 with *p* values larger than 0.05. Their Spearman correlation coefficients with COPD severity except GOLD-IV range from −0.013 to −0.073 with *p* values larger than 0.08.

**Conclusion:**

There is no significant difference in fissure integrity for patients with different levels of disease severity, suggesting that the development of COPD does not change the completeness of pulmonary fissures and incomplete fissures alone may not contribute to the collateral ventilation.

## Introduction

In severe chronic obstructive pulmonary disease (COPD), pulmonary function is largely compromised by hyperinflation [1]–[2]. As an alternative to lung transplantation, the emerging less invasive bronchoscopic lung volume reduction surgery (BLVRS) provides substantial benefit to patients with severe emphysema by reducing hyperinflation without surgically resecting lobes/segments and thus improving airflow [3]–[5]. However, this surgery is not always effective for patients with severe emphysema in particular when significant collateral ventilation exists, which provides passages or channels other than the normal airways for gas exchanges [6]. The existence of collateral ventilation could be helpful for maintaining a certain degree of the pulmonary function within the obstructed areas, but may lead to an ineffective treatment of severe emphysema when inserting a one-way endobronchial valves (EV) in an attempt to occlude segmental bronchi and thus make a specific lobe gradually collapse [6]. Hence, it is desirable to accurately assess the presence (or absence) and extent of collateral ventilation when targeting patients for the most likely success of BLVRS.

In the past, a few investigations [7]–[8] have been performed to identify the pathways for collateral ventilation. Considering that pulmonary fissures subdivide the human lungs into different lobes and air may flow through between lobes, inter-lobar collateral ventilation is particularly studied. Hogg et al. [9] demonstrated in their study that there exists inter-lobar collateral ventilation across major fissures. Sciurba et al. [10] found that forced expiratory volume in one second (FEV1) was improved significantly for patients with complete inter-lobar fissures and heterogeneous emphysema after the EV-based LVRS. Higuchi et al. [11] observed that more inter-lobar collateral ventilation exists in patients with homogeneous emphysema than in those with heterogeneous emphysema. Since pulmonary fissures act as the interface between neighboring lobes, it is natural to suspect whether any characteristic of pulmonary fissure (e.g., fissure integrity) could contribute to the inter-lobar collateral ventilation. However, after an ex vivo evaluation of 23 lungs, Higuchi et al. [11] concluded that there was no significant difference between collateral ventilation and the extent of inter-lobar fissure integrity. Also, Magnussen et al. [12] found that the treatment with an endoscopic tissue sealant was almost equally effective in patients with complete or incomplete fissures. Although Higuchi et al. [11] and Magnussen et al. [12] agreed upon the existence of inter-lobar ventilation, their studies actually did not support the assumption that fissure integrity might contribute to the collateral ventilation. It is notable that fissure integrity was quantified by subjective visual inspection in available investigations [9]–[12]. This is caused by the fact that pulmonary fissures appear as complicated three-dimensional (3D) surfaces and span over multiple CT images, it is difficult and time-consuming in practice for a clinician to accurately assess inter-lobar fissure integrity by manually tracing pulmonary fissures in a slice-by-slice manner. At the same time, only a limited number of exams were involved in these studies [11]–[12]. Whether the conclusions drawn from these investigations are affected by the subjective visual assessment of pulmonary fissure integrity or by limited examinations in their studies is unclear.

In this study, facilitated by a computerized tool in automated fissure integrity assessment and a relatively large diverse dataset consisting of 573 CT exams collected from a COPD study, we propose to investigate whether fissure integrity plays a role in inter-lobar collateral ventilation by studying the relationship between inter-lobar fissure integrity and pulmonary functions. The underlying assumption is that: if incomplete fissures contributes to collateral ventilation, the inter-lobar fissure integrity should be associated with pulmonary function because collateral channels may enable the obstructed lung regions to maintain certain level of function [6], [10], [13]. A detailed description of the method and the experimental results follows.

## Materials and Methods

### A. Study population

A total of 573 CT exams were acquired on different subjects, who enrolled in an NIH-sponsored Specialized Center for Clinically Oriented Research (SCCOR) in COPD at the University of Pittsburgh. Inclusion criteria for enrollment required an age >40 years and at least a 10 pack year history of tobacco use. The SCCOR subjects undergo pre- and post-bronchodilator spirometry and plethysmography, measurement of lung diffusion capacity, impulse oscillometry, a chest CT examination, demographic as well as medical history questionnaires. In order to distinguish COPD from asthma, the post-bronchodilator pulmonary function test (PFT) utilized spirometry to assess the presence of irreversible airflow limitation after the patients inhaled a medication called bronchodilator. All procedures were performed under a University of Pittsburgh Institutional Review Board approved protocol (#0612016) and written informed consent was obtained for each subject. According to the global initiative for chronic obstructive lung disease (GOLD) criteria [14], these subjects (examinations) were classified into five different subgroups, namely non-COPD (222 subjects), GOLD-I (83 subjects), GOLD-II (141 subjects), GOLD-III (63 subjects), and GOLD-IV (64 subjects). The subject demographics of the collected cases to date are shown in Table 1.

**Table 1:** Subject Demographics (*n* = 573).

Parameter	Mean (± std) or count (%)
Sex male	310 (54.1%)
Age	63.9 (±5.4)
Pack years	58.3 (±33.0)
Height(cm)	169.4 (±9.4)
Weight(kg)	80.0 (±15.95)
FEV_1_(litre)	2.15 (±0.94)
FEV_1_/FVC%	60.8 (±17.7)
Five-category classification	
NON-COPD	222 (38.7%)
GOLD I	83 (14.5%)
GOLD II	141 (24.6%)
GOLD III	63 (11.0%)
GOLD IV	64 (11.2%)

*Abbreviations*: FVC – functional vital capacity, FEV_1_ – forced expiratory volume in one second.

### B. Acquisition of thin-section CT examinations

The CT examinations were acquired on a LightSpeed VCT 64-detector scanner (GE Healthcare, Waukesha, WI) with subjects holding their breath at the end of inspiration. The CT data were acquired using a helical technique without contrast at the following parameters: 32×0.625 mm detector configuration, 0.969 pitch,120 kVp tube energy, 250 mA tube current, and 0.4 sec gantry rotation (or 100 mAs). Images were reconstructed to encompass the entire lung field in 512×512 pixel matrix using the GE “bone” kernel at 0.625 mm section thickness and 0.625 mm interval. Pixel dimensions ranged from 0.549 to 0.738 mm, depending on participant body size. This high resolution enables a relatively reliable identification of pulmonary fissure and a relatively accurate quantification of their integrities.

### C. Quantification of pulmonary fissure integrity

Previously, we developed a computerized tool that is capable of: (1) identifying pulmonary fissures [15], (2) classifying pulmonary fissures into major and minor (horizontal) fissures [16], (3) quantifying fissure integrity [16]–[17]. The fissure integrity or completeness was quantified in a geometric space, where the identified fissures and the boundaries between neighboring lobes were modeled as 3D geometric surfaces. By regarding the boundaries between neighboring lobes as the "complete" fissures, which cut through the lungs, the proportion of the fissures depicted on the CT images in terms of area was computed as the fissure integrity. After the application of the tool to the collected CT exams, four experienced image analysts who have sufficient knowledge concerning the depiction of pulmonary fissures on CT images were asked to review the identified fissures and lobes. In case that the fissures and lobes were not properly delineated, manual corrections were made to assure an accurate quantification of fissure integrity until a consensus was reached that the segmentation was adequate. Whereas the performance of the lobe segmentation scheme largely depends on the accuracy of the fissure detection, freehand sketch based manual corrections were only conducted on the automatically detected fissures; thereafter, the automated lobe segmentation scheme [17] was applied to estimate the "complete" fissures by identifying the boundaries between neighboring lobes.

### D. Data analysis

For each subgroup in terms of COPD severity, the average fissure integrities (unit: %) of individual fissures as well as the average fissure integrity at the entire lung level were summarized. We computed the distributions of each individual fissure at six different integrity levels, namely, [0%, 20%], (20%, 40%], (40%, 60%], (60%, 80%], (80%, 90%], and (90%, 100%]. Given the potential error of CT in depicting pulmonary fissures, similar to the studies [10], [12], the integrity level of (90%, 100%] is regarded as "complete". Because of the uneven distributions of CT examinations in terms of COPD severity, we also computed the percentage of the number CT exams at each integrity level for every COPD subgroup. Pearson correlation coefficients were used to assess the association between the fissure integrity and the lung function measures. Here, the post-bronchodilator PFT measurements were used in the correlation analyses, including: (1) FEV1 and (2) FEV1/FVC ratio. In addition, the association between fissure integrity and COPD severity based on our five-category classification were assessed statistically using the Spearman correlation coefficients. A *p*-value less than 0.05 is considered statistically significant. All statistical analyses were performed using Excel (Microsoft Corp., Redmond, WA, USA) and SAS (SAS Institute, Cary, NC, USA).

## Results

The average integrities of individual fissures (i.e., right oblique (major) fissure (ROF), right horizontal (minor) fissure (RHF), and left oblique (major) fissure (LOF)) and all the fissure at the entire lung level (ELF) were summarized in Table 2. The integrities of the right major fissure and the left major fissure were similar (∼82%) regardless of the COPD severity, and the integrity of the right minor fissure was relatively low (∼62%). It can be seen that there is no obvious difference in integrity for the defined subgroups, albeit the fissure integrity for the GOLD-IV subgroup is relatively slightly small. The fissure integrity at the entire lung level was around 78% regardless of the COPD severity. We note that around 19.5% (i.e., 112 scans) of these examinations needed a manual corrections and most of them were classified as moderate and severe COPD.

**Table 2:** Average fissure integrity (unit: %) with standard deviation among sub-groups with different COPD severity.

Fissure Type	Non-COPD	GOLD-I	GOLD-II	GOLD-III	GOLD-IV
right major (oblique) fissure	81.8±1.3	82.4±1.0	81.8±1.7	82.8±1.0	80.2±1.4
right minor (horizontal) fissure	62.6±6.2	61.8±6.2	62.1±5.8	62.2±5.8	62.3±4.6
left major (oblique) fissure	82.0±1.5	83.2±1.1	81.7±1.4	82.0±1.8	78.5±1.5
All fissures at the entire lung	78.0±1.2	78.6±0.9	78.2±1.3	78.5±1.2	76.4±1.1

**Figure 1:**
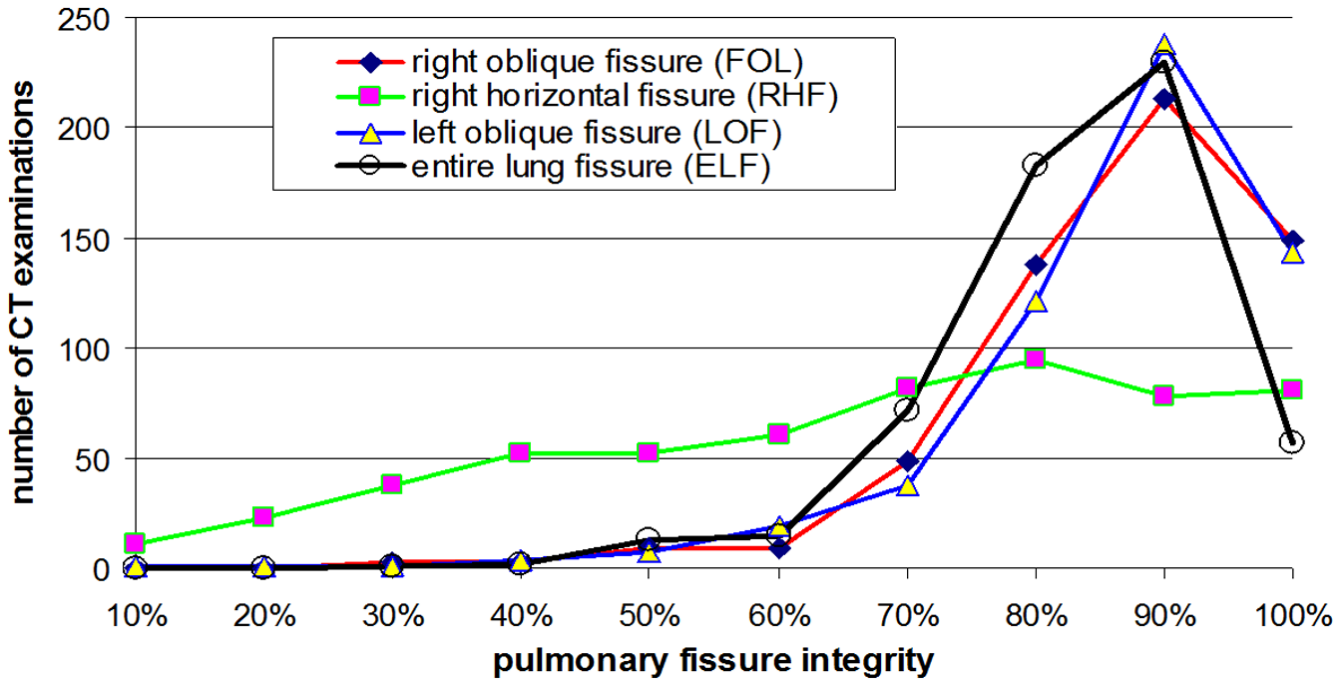
Distribution of the integrity levels of pulmonary fissures in all COPD patients.

The distributions of individual fissures (i.e., ROF, RHF, LOF, and ELF) at different integrity levels were displayed in Figure 1. ROF and LOF had similar distributions at different integrity levels. In terms of COPD severity and fissure integrity, the percentage distributions of the collected exams were summarized separately in Table 3–Table 6. Around 75% of the enrolled subjects, regardless of their COPD severity, have an integrity level larger than 90% for both ROF and LOF. For each type of pulmonary fissure, the percentage-based distributions of the CT exams among different COPD subgroups were similar in integrity level.

**Table 3:** Distribution (number/percentage (%)) of the CT examinations in terms of right oblique fissure (ROF) integrity and COPD severity.

Integrity of ROF	Non-COPD	GOLD-I	GOLD-II	GOLD-III	GOLD-IV	All Groups
[0, 20%]	0/0%	0/0%	0/0%	0/0%	0/0%	0/0%
(20%, 40%]	3/1.4%	0/0%	3/2.1%	0/0%	0/0%	6/1.1%
(40%, 60%]	5/2.3%	3/3.6%	3/2.1%	2/3.2%	5/7.8%	18/3.1%
(60%, 80%]	74/33.3%	26/3.1%	45/31.9%	20/31.7%	22/34.4%	187/32.6%
(80%, 90%]	82/36.9%	35/42.2%	52/36.9%	22/34.9%	22/34.4%	213/37.2%
(90%, 100%]	58/26.1%	19/22.9%	38/27.0%	19/30.2%	15/23.4%	149/26.0%

**Table 6:** Distribution (number/percentage (%)) of the CT examinations in terms of entire lung fissure (ELF) integrity and COPD severity.

Integrity of FEL	Non-COPD	GOLD-I	GOLD-II	GOLD-III	GOLD-IV	All Groups
[0, 20%]	0/0%	0/0%	0/0%	0/0%	0/0%	0/0%
(20%, 40%]	1/0.5%	0/0%	2/1.4%	0/0%	0/0%	3/0.5%
(40%, 60%]	13/5.9%	3/3.6%	6/4.3%	3/4.8%	3/4.7%	28/4.9%
(60%, 80%]	100/45.0%	36/43.4%	59/41.8%	27/42.9%	33/51.6%	255/44.5%
(80%, 90%]	82/36.9%	38/45.8%	60/41.6%	24/38.1%	26/40.6%	230/40.1%
(90%, 100%]	26/11.7%	6/7.2%	14/9.9%	9/14.2%	2/3.1%	57/10.0%

**Table 5:** Distribution (number/percentage (%)) of the CT examinations in terms of left oblique fissure (LOF) integrity and COPD severity.

Integrity of LOF	Non-COPD	GOLD-I	GOLD-II	GOLD-III	GOLD-IV	All Groups
[0, 20%]	1/0.5%	0/0%	1/0.7%	0/0%	0/0%	2/0.3%
(20%, 40%]	2/0.9%	0/0%	0/0%	2/3.2%	1/1.6%	5/0.9%
(40%, 60%]	10/4.5%	3/3.6%	7/5.0%	2/3.2%	4/6.2%	26/4.5%
(60%, 80%]	59/26.6%	20/24.1%	40/28.4%	14/22.2%	26/40.6%	159/27.8%
(80%, 90%]	88/39.6%	38/45.8%	59/41.8%	28/44.4%	25/39.1%	238/41.5%
(90%, 100%]	62/27.9%	22/26.5%	34/24.1%	17/27.0%	8/12.5%	143/25.0%

The correlation coefficients between the integrities of individual fissures and the pulmonary function measures (i.e., FEV1 and FEV1/FVC) as well as disease severity (i.e., GOLD level) were listed in Table 7, suggesting that pulmonary fissures integrity does not affect pulmonary function and disease severity.

**Table 7:** Correlations (Pearson correlation coefficients) between fissure integrity and PFT measures.

PFT measures	right major fissure	right minor fissure	left major fissure	All fissures
FEV1	0.004494 (*p* = 0.9145)	0.007788 (*p* = 0.8524)	0.097132 (*p* = 0.0200)	0.024726 (*p* = 0.5547)
FEV1/FVC	0.027496 (*p* = 0.5112)	0.048561 (*p* = 0.2458)	0.103589 (*p* = 0.7279)	0.05562 (*p* = 0.9562)
COPD severity	−0.01391 (*p* = 0.7397)	−0.01917 (*p* = 0.6470)	−0.07373 (*p* = 0.0778)	−0.01948 (*p* = 0.6417)

## Discussion

In this study, we investigated whether inter-lobar fissure integrity could serve a biomarker for collateral ventilation by studying its association with lung function. The strength of this study is the involvement of a large diverse dataset acquired using the same protocol and the objective quantification of inter-lobar fissure integrity. In Higuchi et al.'s study [11], they only evaluated 23 lungs with severe emphysema and classified the inter-lobar fissure into four categories by visually examining the lungs after deflation. In Magnussen et al.'s study [12], the major and minor fissures were independently inspected by two readers and classified into two categories, namely complete (integrity ≥90%) and incomplete (<90%). Sciurba et al. [10] defined the complete fissures as those with an visual integrity ≥90% as well. In contrast, we used our in-house developed computer algorithms to automatically process the collected exams and quantify the fissure integrities objectively. In particular, the segmentation results were validated visually by four image analysts. The image analysts have been actively involved in a number of projects related to lung CT image analysis and have sufficient knowledge concerning the radiologic depiction of pulmonary fissures in CT examinations.

Our study demonstrated that there was no obvious variation in fissure integrity for the subgroups with different COPD severity. Accordingly, we do not find any association between fissure integrity and pulmonary function measures, such as FEV1 and FEV1/FVC (Table 7). This finding highlights two points: (1) development of COPD does not affect the integrity of pulmonary fissures and (2) incomplete fissures may not contribute to collateral ventilation. The later one is consistent with previous investigations [11]–[12]. However, we have to note that we cannot make any conclusion that emphysema damage or lung function change would not affect other morphological characteristics (e.g., geometric shape and/or thickness) of pulmonary fissures. Nevertheless, collateral ventilation could be very complex and affected by a number of factors. In this study, we proposed to indirectly assess the impact of fissure integrity on collateral ventilation by assuming that collateral channels may help to maintain certain level of function [6], [10], [13]. Hence, we believe that additional efforts may be needed for a concrete verification, but they are out of the scope of this study.

At the same time, this study provided a relatively comprehensive and objective analysis of the prevalence of pulmonary fissure integrity among COPD patients. As our results demonstrated, pulmonary fissures are often incomplete. The average fissure integrity is around 78%, regardless of the COPD severity. Specifically, the oblique fissure typically has an integrity level of around 82%, and the horizontal fissure has an integrity level of around 62%. With consideration of potential errors in integrity quantification, fissures with an integrity larger than 90% are regarded as "complete" fissures. As a result, more than 90% of the patients involved in this study had incomplete fissures. This number is similar to the result (i.e., 95% of subjects have incomplete fissures) reported by Mahmut et al. [18]. Typically, the oblique fissures, no matter in the right or the left lung, have an integrity level larger than 60%. As compared to the oblique fissures, the horizontal fissure has a wide range of variety in integrity (Table 4), and a certain number of subjects have an integrity level of less than 40%. Approximately, 75% of the involved subjects have incomplete oblique fissures and 84% of the involved subjects have incomplete horizontal fissures. Half of these subjects have an integrity level from 40%–80% for the horizontal fissures, and around 70% of these subjects have an integrity level from 60–80% for the oblique fissures. In the past, a couple of investigations [19]–[28] have been performed to investigate the prevalence of fissure integrity as well as their potential clinical implications, albeit their results were based on subjective visual assessment. For example, Berkmen et al [16] observed incomplete fissures in 42.2% of the subjects. When the fissures in the left and right lungs were studied separately, 48% of the right major (oblique) fissures and 43% of the left fissures were assessed as incomplete by Aziz et al [22]. When the fissure completeness was classified into four broad categories of completeness, Matsuoka et al [28] reported that the fissures were complete in 29%, slightly incomplete in 44%, incomplete in 17%, and considerably incomplete in 10% of the 41 imaged subjects. Our computer results are consistent with these studies but with an obviously large dataset and objective quantification.

**Table 4:** Distribution (number/percentage (%)) of the CT examinations in terms of right horizontal fissure (RHF) integrity and COPD severity.

Integrity of RHF	Non-COPD	GOLD-I	GOLD-II	GOLD-III	GOLD-IV	All Groups
[0, 20%]	15/6.7%	8/9.6%	5/3.5%	3/4.8%	3/4.7%	34/5.9%
(20%, 40%]	33/14.9%	10/12.0%	27/19.1%	13/20.6%	7/10.9%	90/15.7%
(40%, 60%]	41/18.5%	15/18.1%	30/21.3%	9/14.3%	18/28.1%	113/19.7%
(60%, 80%]	66/29.7%	29/34.9%	39/27.7%	21/33.3%	22/34.4%	177/30.9%
(80%, 90%]	34/15.3%	9/10.9%	17/12.1%	10/15.9%	8/12.5%	78/13.6%
(90%, 100%]	33/14.9%	12/14.5%	23/16.3%	7/11.1%	6/9.4%	81/14.1%

Although we tried to analyze the association between fissure integrity and collateral ventilation, we did not measure the collateral ventilation directly but analyzed the impact of fissure integrity on pulmonary function. This is primarily caused by the fact that it is extremely difficult to measure the collateral ventilation. For example, Higuchi et al. [11] measured the collateral ventilation by subjectively assessing how easy it was to ventilate a non-intubated lobe after explanation. As Cetti et al. [6] pointed out, this may oversimplify the collateral ventilation. Alternatively, Morrell et al. [29] proposed to measure collateral ventilation by occluding a lobe or segment with a balloon catheter under the guidance of a bronchoscope; however, their measurement showed the existence of collateral ventilation even for normal segment, suggesting the potential unreliability of this method. In particular, Sciurba et al. analyzed both fissure integrity and emphysema heterogeneity as a surrogate for interlobar collaterals in their study [10]. Hence, in this study, considering that collateral channels enable the obstructed lung regions to maintain certain level of function, we proposed to assess the association between fissure integrity and lung function. We are aware that this strategy may oversimplify the complexity of collateral ventilation and could be a limitation or drawback of this study. In an individual with an incomplete fissures surrounded or breached by emphysema, the probability for interlobar collateral ventilation may be much greater, because the emphysema (or absence of parenchyma) could serve as a conduit to permit airflow between neighboring lobes. Therefore, fissure integrity alone may not be sufficient for inferring its impact on pulmonary function and collateral ventilation. It may be desirable to develop an integrative model that incorporates other factors, such as the disease status near the incomplete fissures, for a comprehensive investigation of the role of fissure integrity in pulmonary function and disease progression.

## Conclusions

We investigated the prevalence of incomplete pulmonary fissures in COPD patients and the impact of fissure completeness on pulmonary function using a relatively large, diverse dataset consisting of 573 CT exams. The integrities of the pulmonary fissures depicted on these CT images were quantified objectively using our in-house developed algorithms. Our experimental results show that around 90% of the subjects have incomplete fissures and the right and left major fissures have similar integrity, while the right minor fissure has a relatively low integrity. We in particular found that there was no obvious difference in fissure integrity for the subgroups with different COPD severity. In other words, the development of COPD does no affect the fissure integrity and fissure integrity alone may not contribute to the collateral ventilation.
